# Does a Large-Ear Wheat Cultivar Benefit More from Elevated CO_2_ than a Multiple-Ear Wheat for the Utilization of Non-Structural Carbohydrates?

**DOI:** 10.3390/plants15030419

**Published:** 2026-01-30

**Authors:** Yuting Li, Han Yu, Yuqiao Xia, Zhenhua Zhang, Qiufeng Wang, Bingjian Sha, Han Xue

**Affiliations:** 1 Jiangsu Key Laboratory for Bioresources of Saline Soils, Jiangsu Synthetic Innovation Center for Coastal Bio-Agriculture, Jiangsu Provincial Key Laboratory of Coastal Bio-Agriculture, Jiangsu Provincial Key Laboratory of Coastal Wetland Bioresources and Environmental Protection, School of Wetland, Yancheng Teachers University, Yancheng 224002, China; 2 The School of Agriculture and Environment, The University of Western Australia, Crawley, WA 6009, Australia; 3 Institute of Environment and Sustainable Development in Agriculture, Key Laboratory of Agro-Environment and Climate Change of Agriculture Ministry, Chinese Academy of Agricultural Sciences, Beijing 100081, China

**Keywords:** elevated CO_2_, non-structural carbohydrate, winter wheat cultivars, ear types

## Abstract

The effects of elevated CO_2_ concentration (eCO_2_) on the utilization of carbohydrates in wheat cultivars with different ear types remain poorly understood, despite the critical role of wheat ears as major carbohydrate sinks and the importance of ear type as a key growth trait influencing crop yield. In this study, a free-air CO_2_ enrichment (CAAS-FACE) facility was utilized to investigate the effects of eCO_2_ on the non-structural carbohydrate (NSC) utilization in two wheat cultivars: a large-ear cultivar (cv. Shaanhan 8675) and a multiple-ear cultivar (cv. Triumph). The findings demonstrated that under eCO_2_ conditions, Shaanhan 8675 exhibited enhanced NSC availability and more efficient remobilization from vegetative organs to grains. This improvement was associated with sustained photosynthetic activity in the leaves during the grain-filling period, which contributed to better grain filling. Consequently, both grain NSC accumulation and kernel weight were significantly increased in Shaanhan 8675 under eCO_2_. In contrast, the grain NSC accumulation in Triumph was constrained by limited translocation of NSC from the stem and ear to the grain under eCO_2_ environment. Overall, our findings suggest that CO_2_ enrichment has a pronounced positive effect on NSC utilization in large-ear wheat cultivars. These results contribute to strategies aimed at ensuring stable and high wheat yields under future climatic conditions.

## 1. Introduction

By 2050, the expanding global population will reach 9.1 billion, which demands increased crop production to ensure global food security [1]. Wheat (*Triticum aestivum* L.) is one of the most important staple crops in the human diet, directly contributing approximately 20% of the total caloric and protein intake worldwide [2]. In China, wheat cultivation is predominantly concentrated in the central and eastern regions, with approximately 18.5 million hm^2^ accounting for 70.1% of the country’s total wheat planting area dedicated to its production. However, numerous studies have highlighted the potential impacts of global climate change on wheat productivity [3,4]. According to projections by the IPCC, they predicted that atmospheric CO_2_ concentrations are expected to reach 550 µmol·mol^−1^ by 2050 and up to 1020 µmol·mol^−1^ by 2100 [4], which will inevitably influence the production of wheat.

As a typical C3 crop, wheat exhibits high sensitivity to climatic and environmental fluctuations [5]. Since atmospheric carbon dioxide (CO_2_) serves as the substrate for Rubisco-catalyzed carboxylation in plants, variations in climate, particularly rising CO_2_ levels, are expected to have direct impacts on wheat production [3]. The effects of elevated CO_2_ concentration (eCO_2_) on wheat grain yield, grain quality, and plant physiological responses have been extensively investigated [4]. Furthermore, previous studies on CO_2_ enrichment have revealed significant interspecific variation in plant biomass accumulation and grain protein response to eCO_2_ [6]. This variation can be attributed to differences in photosynthetic capacity and carbohydrate utilization efficiency among genotypes with diverse genetic backgrounds [7]. Enhanced biomass production, particularly when accompanied by an optimized source–sink relationship, has been proposed as a key driver for achieving substantial increases in wheat grain yield [7]. When grown under eCO_2_ environment, C_3_ crops such as wheat can significantly increase grain yield if the carbon supply (photosynthetic capacity) and sink capacity (storage strength) are well coordinated. Specifically, this synergy involves enhanced carbon assimilation during the vegetative stage and improved sink strength during reproductive growth [8,9,10]. In wheat, the primary carbon sink is grain during the middle and late stages of growth and development; hence, the ear is a crucial carbon sink, especially during the later growth stage [11]. Numerous studies have also demonstrated that efficient carbohydrate utilization enables plants to mitigate photosynthetic downregulation under prolonged exposure to an elevated CO_2_ environment [12]. Therefore, future agricultural systems under elevated atmospheric CO_2_ will require cultivars with superior photosynthetic efficiency, enhanced carbon assimilation capacity, and effective carbon redistribution mechanisms. To address this need, we selected two winter wheat cultivars representing contrasting ear types: large-ear- and multiple-ear-type winter wheat cultivars to investigate whether genotypes with stronger reproductive sinks can achieve higher kernel weights through more efficient utilization of non-structural carbohydrates under elevated CO_2_ conditions.

In detail, we hypothesized that elevated carbon dioxide concentrations would enhance the photosynthetic efficiency of wheat leaves, thereby promoting greater accumulation of non-structural carbohydrates (NSC) within the plant. Large-ear wheat cultivars are expected to exhibit stronger “sink strength” during the grain-filling period, which could facilitate the translocation and reallocation of NSC stored in various vegetative organs to the developing grains. This enhanced sink capacity may, to some extent, allow for functional leaves to maintain a higher photosynthetic rate during the grain-filling stage, leading to increased NSC accumulation in the grains and, consequently, improved kernel weight. The findings of this study will contribute to the theoretical understanding of how climate resources can be effectively utilized in crop production, supporting strategies for ensuring stable and high wheat yields under future CO_2_-enriched environments.

## 2. Materials and Methods

### 2.1. Experimental Site and Growing Conditions

**Figure 1:**
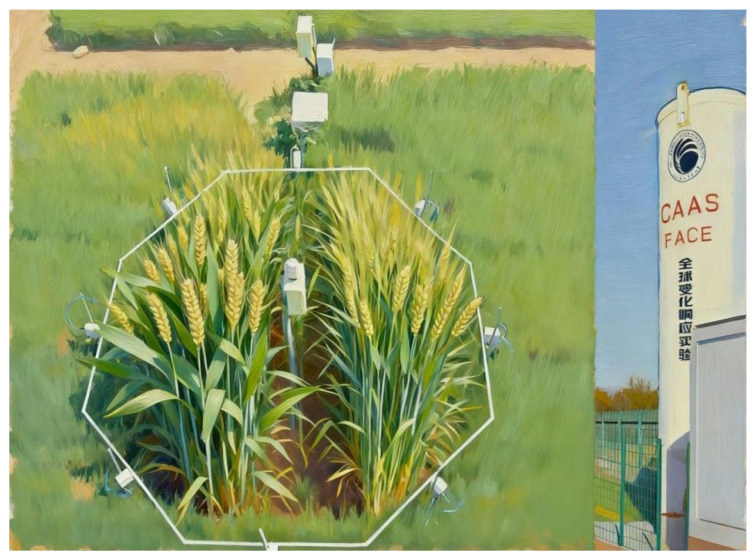
Mini-free air carbon dioxide enrichment system of Chinese Academy of Agricultural Sciences (CAAS-FACE system) in Changping, Beijing, China.

This study was conducted at the wheat–maize rotation CAAS-FACE system in Changping (40°13′ N, 116°14′ E), Beijing, China. This system site is on a clay loam (0–0.20 m soil) with a pH (soil–water ratio of 1:5) of 8.4 and contains 14.10 g·kg^−1^ organic C, 0.82 g·kg^−1^ total N, 19.97 mg·kg^−1^ available phosphorus, and 79.77 mg·kg^−1^ ammonium acetate extractable potassium. The CAAS-FACE system includes six elevated CO_2_ (550 ± 17 µmol·mol^−1^) and six ambient CO_2_ octagonal plots (415 ± 16 µmol·mol^−1^), each with a diameter of 4 m. The carbon dioxide concentrations of each plot were measured by sensors (Vaisala, Vantaa, Finland) at the center of each octagonal plot, during the experimental season. The experimental plots were separated by at least 14 m to minimize cross-contamination of CO_2_ between experimental treatments. Cross-contamination was minimal, according to a comparison of the CO_2_ concentrations in ambient plots with and without the release of CO_2_ gas to elevated plots (Figure 1).

**Figure 2:**
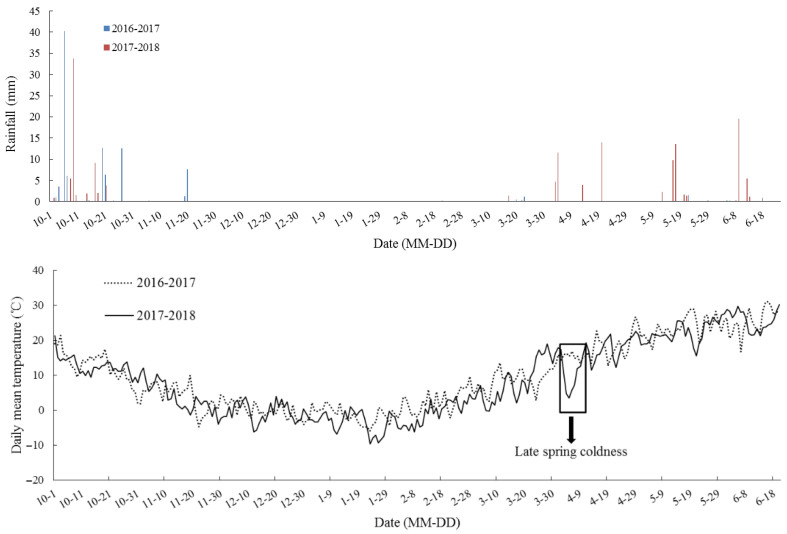
Rainfall (mm) and daily temperature (°C) at the wheat–maize rotation CAAS-FACE system in Changping from the sowing of winter wheat until maturity in 2016–2018, two continuous contrasting experiment years. The downward arrow indicates late spring coldness in 2018.

In the eCO_2_ treatment, carbon dioxide exposure commenced one week after sowing and terminated at maturity, and CO_2_ was maintained at 550 ± 17 µmol·mol^−1^ from 6:00 to 19:00 h. The experiments were carried out during two growing seasons, from 2016 to 2018. According to the climatic database, the rainfall of two winter wheat growing seasons from 2016 to 2018 was 98.1 mm and 149.4 mm, respectively, and the corresponding mean temperature was 9.7 °C and 8.5 °C at this location (Figure 2).

In this experiment, a split-plot randomized block design was adopted with 3 replications; CO_2_ concentration was the main treatment (aCO_2_, 415 ± 16 mol·mol^−1^ and eCO_2_, 550 ± 17 mol·mol^−1^) and winter wheat cultivar was the sub-treatment.

### 2.2. Plant Material and Fertilization

In this research, *Triticum aestivum* L. cv. Shaanhan 8675 and Triumph were chosen for this research. Shaanhan 8675 is a wheat cultivar developed by the Shaanxi Provincial Wheat Research Institute, with its parental cross combination derived from Changwu 131 and Xizhi 81206. Owing to its excellent yield performance, this cultivar has been extensively utilized in wheat cross-breeding programs. Triumph is an early-introduced American wheat cultivar whose genetic background harbors desirable agronomic traits, such as strong stress tolerance and large population capacity. Owing to its excellent comprehensive agronomic traits, this cultivar has become one of the core genetic resources for wheat breeding in China. According to the ear traits and the ratio of harvest index (HI), Shaanhan 8675 is regarded as a large-ear cultivar (Table 1). In contrast, the ear size of Triumph is relatively small as a multiple-ear type when compared with Shaanhan 8675 (Table 1).

Each plot area was 6 m^2^. The seeding rate was determined according to the 1000-grain weight of each cultivar to ensure the basic seedlings of each cultivar at 300 plants per square meter. The drill sowing method was adopted to sow 6 rows in each plot with a row spacing of 20 cm. Granular urea (N, 46%), diammonium phosphate (N:P_2_O_5_ = 13%:44%), and potassium chloride (K_2_O, 60%) were applied as basal fertilizer at equal rates of 100 kg N·hm^−2^, 165 kg P_2_O_5_·hm^−2^, and 90 kg K_2_O·hm^−2^. At the stage of jointing on 28 April 2017 and 2018, granular urea at a rate of 100 kg N·hm^−2^ was applied as a side dressing. Irrigation was applied twice during the whole growing season of winter wheat: the overwintering irrigation at a quantity of 750 m^3^·hm^−2^ on 23 November 2016 and 2017, and the spring irrigation at a quantity of 750 m^3^·hm^−2^ of water at the jointing stage after side dressing fertilization.

### 2.3. Crop Measurements

#### 2.3.1. Light-Saturated Maximum Gross CO_2_ Assimilation Rate

Light-saturated net CO_2_ assimilation rate (*A_g,max_*, μmol·m^−2^s^−1^) measurements were performed on the latest fully expanded leaf from three individual plants of each treatment on sunny days between 9:00 and 11:30 a.m. at the jointing, anthesis, and grain-filling stages. The measurements were taken using an LI-6400 portable photosynthesis system (LiCor-6400XT, LI-Cor, Lincoln, NE, USA). Settings in the leaf chamber are as follows: CO_2_ concentration was set at 415 μmol·mol^−1^ in aCO_2_ treatments and 550 μmol·mol^−1^ in eCO_2_ treatments, respectively; the photosynthetic active radiation was set to 1200 μmol·m^−2^s^−1^; the temperature inside the leaf chamber was set to a temperature consistent with the ambient atmospheric temperature.

#### 2.3.2. NSC Measurement and Calculation

Plants were sampled at the anthesis stage, destructive sampling was performed on a 20 cm^2^ area of plants within each CO_2_ treatment, and after removing the soil from the roots, all the plants were separated according to the number of single stems. The number of plants and stems was recorded and converted to the number of plants and stems per unit area, and then 20 plants were taken according to the ratio of plants with different numbers of single stems to the total number of plants in the sample as the plant samples for the NSC measurement and calculation. Plant samples were separated into the leaf, stem, and ear (glumes and ear-stalks), and deactivated at 150 °C for 30 min, then dried at 80 °C to a constant weight. Samples were then ground and filtered through a 0.5 mm sieve to prepare the powdered material for NSC measurement. Soluble sugar contents were analyzed using an anthrone reagent according to the method from Mustroph et al. (2006) [13]. Sucrose content (SC) and starch content were measured using a resorcinol reagent and a 3,5-dinitrosalicylic acid colorimetry reagent according to the procedures described by Wang et al. (2019) [14] and the adoption of a multimode microplate reader (Infinite 200 PRO Nano Quant, Tecan) to measure spectrophotometrically. The starch content was calculated as mg g^−1^ dry weight [15]. In this study, the sums of sugars and starch concentrations were estimates of NSC [16]. The transfer of organ NSC parameters were calculated according to the following equations [16,17]:

(1) Organ NSC accumulation (TM_NSC_ g·m^−2^) = Organ dry weight × Organ NSC content

(2) Organ NSC partitioning index (%) = (Organ NSC accumulation/Plant NSC accumulation) × 100%

(3) Apparent transferred mass of organ NSC (ATM_NSC_, g·m^−2^) = Organ NSC accumulation at anthesis − Organ NSC accumulation at maturity

(4) Apparent ratio of transferred NSC from organ (AR_N__SC_,%) = (Apparent transferred mass of organ NSC/Organ NSC accumulation at anthesis) × 100

(5) Apparent contribution of transferred organ NSC to grain yield (AC_NSC_) = Apparent transferred mass of organ/Dry grain weight × 100

#### 2.3.3. Measurement of Kernel Number and Kernel Weight

Wheat plant samples were collected at the ripening stage from six rows of 3 m length in the center of each plot were harvested at ground level to minimize edge effects. Separating all wheat ears from the plants, and all ears were dried in an oven at 105 °C for 30 min, and then at 80 °C until constant weight. Twenty ears were randomly selected from each experimental plot at the maturity stage, and three kernel-related traits were determined for each sampled ear: total kernel number, number of degenerated kernels, and number of fully filled kernels. The kernel number in each ear of these twenty individuals was recorded, and the kernel number per ear of every treatment was the mean kernel number for each ear of the 20 harvested individuals. After this, all the ears were threshed, and kernels were soaked in tap water of 1.00 specific gravity, and the number of degenerated kernels and filled kernels was recorded. All kernels were weighed after drying at 70 °C to constant weight, and the weight of each kernel and the kernel weight per ear were recorded.

### 2.4. Data Statistical Processing

Statistical analyses for all data generated in this study were performed using SPSS 18.0 version data statistical software and Excel 2016. The experiment was designed as a split-plot with the whole plots arranged in randomized complete blocks; levels of CO_2_ (ambient or elevated CO_2_) were the whole-plot treatment, and wheat cultivars were the split-plot treatment. A general linear model was used to estimate the main effects of CO_2_ and cultivars, as well as their interactions. ANOVA was used to test for statistical significance to determine differences between treatment means. The least significant difference (LSD) at *p* < 0.05 was used to compare the means between CO_2_ concentration and cultivar treatments.

## 3. Results

### 3.1. Effects of Elevated CO_2_ on the Kernels of the Ear at the Ripening Stage

Elevated CO_2_ reduced the amount of degenerated kernels per ear of Shaanhan 8675 by 27.7% and 22.9% (*p* < 0.05) across two years, but increased filled kernels per ear by 9.3% and 9.8% (*p* < 0.05) (Table 2). However, in 2018, eCO_2_ increased the degenerated kernels per ear of Triumph while decreasing the filled kernels per ear by 12.1% (*p* < 0.05) (Table 2). When CO_2_ concentration increased to 550 µmol·mol^−1^, Shaanhan 8675 produced fewer degenerated kernels per ear than Triumph, while the results for filled kernels per ear were the opposite (Table 2). CO_2_ × cultivar interaction was detected for the kernel weight per ear and kernel weight per square meter. Elevated CO_2_ increased the kernel weight per ear and kernel weight per square meter of Shaanhan 8675 by an average of 13.4% and 25.6% (*p* < 0.05) across two years. However, eCO_2_ had no significant effect on kernel weight per ear or kernel weight per square meter of Triumph. Therefore, under the eCO_2_ treatment, these parameters of Shaanhan 8675 were an average of 45.3% and 38.7% (*p* < 0.05) higher than Triumph throughout the course of two years (Table 2).

### 3.2. Light-Saturated Maximum Gross Photosynthetic Rate

**Figure 3:**
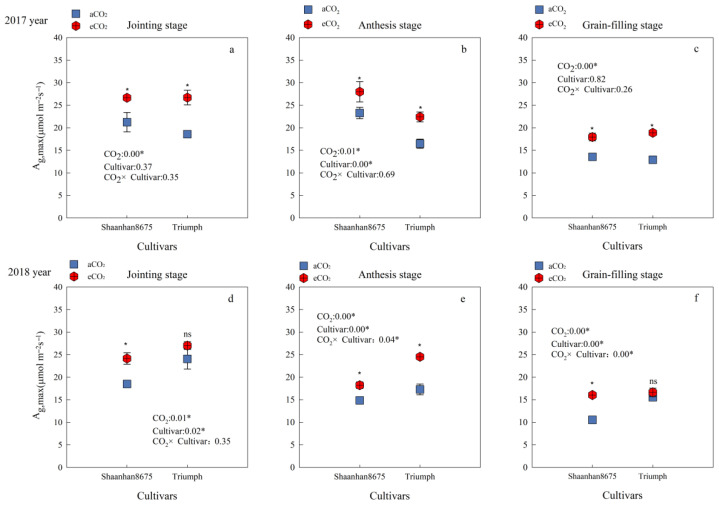
Effects of elevated CO_2_ on the *A_g,max_* (μmol·m^−2^s^−1^) at three key growth stages of two winter wheat cultivars grown under either ambient CO_2_ (aCO_2_, 415 µmol·mol^−1^) or elevated CO_2_ (eCO_2_, 550 µmol·mol^−1^). Measurements were carried out on the last fully expanded leaf. Data represent the mean of three plants from each cultivar pot ± SD (standard error) bars. ANOVA results are also shown with * and ns indicating *p* < 0.05 and no significance, respectively. (**a**) 2017 year Jointing stage, (**b**) 2017 year Anthesis stage, (**c**) 2017 year Grain-filling stage, (**d**) 2018 year Jointing stage, (**e**) 2018 year Anthesis stage, (**f**) 2018 year Grain-filling stage.

Averaged across the jointing, anthesis, and grain-filling stages, eCO_2_ increased the *A_g_*_,_*_max_* of Shaanhan 8675 by 26.1% and 35.2%, respectively, over the two years. eCO_2_ increased the *A_g_*_,_*_max_* of Triumph by 42.1% (*p* < 0.05), averaged across the three key growth stages in 2017. However, in 2018, the *A_g_*_,_*_max_* of Triumph increased by 41.9% (*p* < 0.05) only at the anthesis stage (Figure 3e). Cultivar differences showed that in 2017, the *A_g_*_,_*_max_* of Shaanhan 8675 was higher than that of Triumph at the anthesis stage under eCO_2_ treatments (Figure 3b). However, the results were reversed in 2018 (Figure 3e).

### 3.3. NSC Accumulation and Allocation of Different Plant Tissues at the Anthesis Stage

There were significant interactions between CO_2_ and the cultivar for TM_NSC_ (Table 3). Except for the TM_NSC_ in the ear of Shaanhan 8675 in 2018, eCO_2_ significantly increased the TM_NSC_ of Shaanhan 8675 organs in both years. As for Triumph, eCO_2_ increased TM_NSC_ in the leaf by 43.5% (*p* < 0.05) in 2018 only (Table 3). When CO_2_ increased to 550 µmol·mol^−1^, TM_NSC_ in the stem and ear of Shaanhan 8675 was significantly higher than that of Triumph across two years.

Elevated CO_2_ significantly reduced PI_NSC_ in the stem but increased PI_NSC_ in the ear of Shaanhan 8675 in 2017. Elevated CO_2_ significantly increased the PI_NSC_ in the leaf but decreased the PI_NSC_ in the ear of Triumph in 2018. Cultivar difference results showed that the PI_NSC_ of Triumph leaf was higher than that of Shaanhan 8675 under eCO_2_ treatments in both years, whereas the PI_NSC_ of ear was opposite.

### 3.4. Sucrose Content and the Ratio of Sucrose to NSC of Organs at the Anthesis Stage

Elevated CO_2_ significantly increased the SC and the ratio of sucrose to NSC (RS) of Shaanhan 8675 organs in both years (Table 4). As for Triumph, eCO_2_ significantly decreased the SC and RS in the leaf and the stem in 2017. However, elevated CO_2_ increased the RS in the ears of Triumph across two experimental seasons (Table 4). The results of cultivar differences indicate that, when CO_2_ increased to 550 µmol·mol^−1^, except for the RS of the ears in 2018, the SC and RS of Shaanhan 8675 organs were significantly higher than those of Triumph in both years (Table 4).

### 3.5. Effects of Elevated CO_2_ on SPS Activity in Leaves at the Anthesis Stage

**Figure 4:**
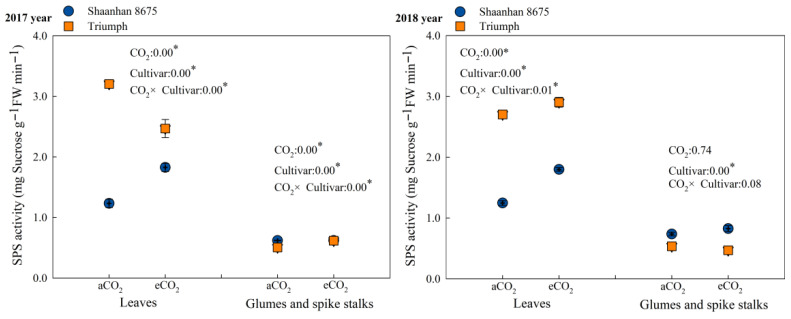
Effects of elevated CO_2_ on SPS (sucrose phosphate synthase) activity at the anthesis stage in the leaves of two winter wheat cultivars grown under either ambient CO_2_ (aCO_2_, 415 µmol·mol^−1^) or elevated CO_2_ (eCO_2_, 550 µmol·mol^−1^). Data represent the mean of three plants from each cultivar pot ± SD (standard error) bars. ANOVA results are also shown with * indicating *p* < 0.05.

Elevated CO_2_ concentration upregulated the activity of sucrose phosphate synthase (SPS) (Figure 4) and significantly increased sucrose content in the leaves of Shaanhan 8675 at the anthesis stage (Table 3). This indicates that elevated CO_2_ promoted photosynthetic carbon assimilation in Shaanhan 8675 leaves during the flowering period and enhanced the storage of photosynthetic carbon assimilates in the form of sucrose. Consequently, this led to increased carbon export to sinks, thereby facilitating panicle development and reducing the number of abortive spikelets. In contrast, elevated CO_2_ exerted a downregulatory effect on SPS activity in the leaves of Triumph (Figure 4). This promoted sucrose degradation and starch synthesis, resulting in decreased sucrose content in Triumph leaves (Table 3). As a result, the export of photosynthetic carbon assimilates to sinks was reduced, which was unfavorable for panicle development.

### 3.6. Effects of Elevated CO_2_ on the Contribution of NSC in Plant Tissues to Grain Yield

**Figure 5:**
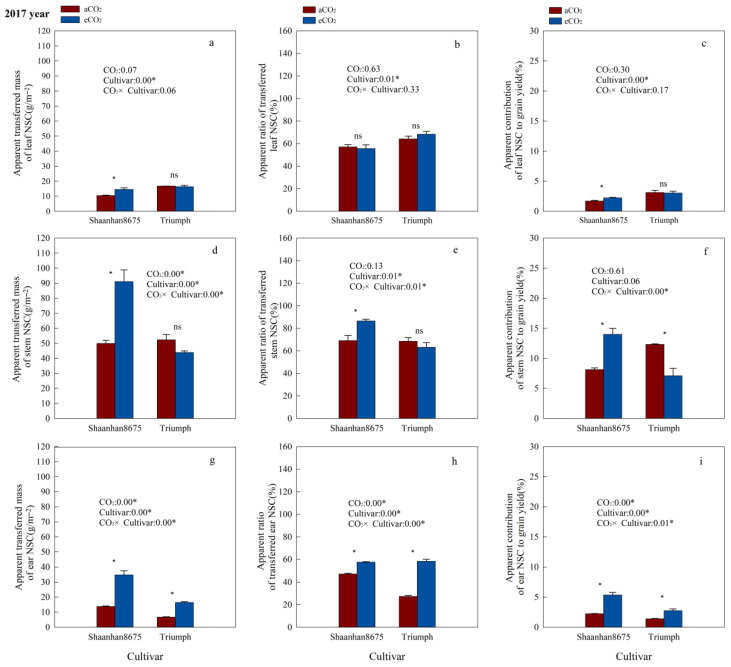
Effects of elevated CO_2_ on the ATM_NSC_ (apparent transferred mass of organ NSC), AR_NSC_ (apparent ratio of transferred NSC from organ), and AC_NSC_ (apparent contribution of transferred organ NSC to grain yield) of organs in two winter wheat cultivars grown under either ambient CO_2_ (aCO_2_, 415 µmol·mol^−1^) or elevated CO_2_ (eCO_2_, 550 µmol·mol^−1^) in 2017. Data represent the mean of three plants from each cultivar pot ± SD (standard error) bars. ANOVA results are also shown, with * indicating *p* < 0.05 and ns indicating no significance. (**a**) Apparent transferred mass of leaf NSC (g/m^−2^); (**b**) Apparent ratio of transferred leaf NSC (%); (**c**) Apparent contribution of leaf NSC to grain yield (%); (**d**) Apparent transferred mass of stem NSC (g/m^−2^); (**e**) Apparent ratio of transferred stem NSC (%); (**f**) Apparent contribution of stem NSC to grain yield (%); (**g**) Apparent transferred mass of ear NSC (g/m^−2^); (**h**) Apparent ratio of transferred ear NSC (%); (**i**) Apparent contribution of ear NSC to grain yield (%).

**Figure 6:**
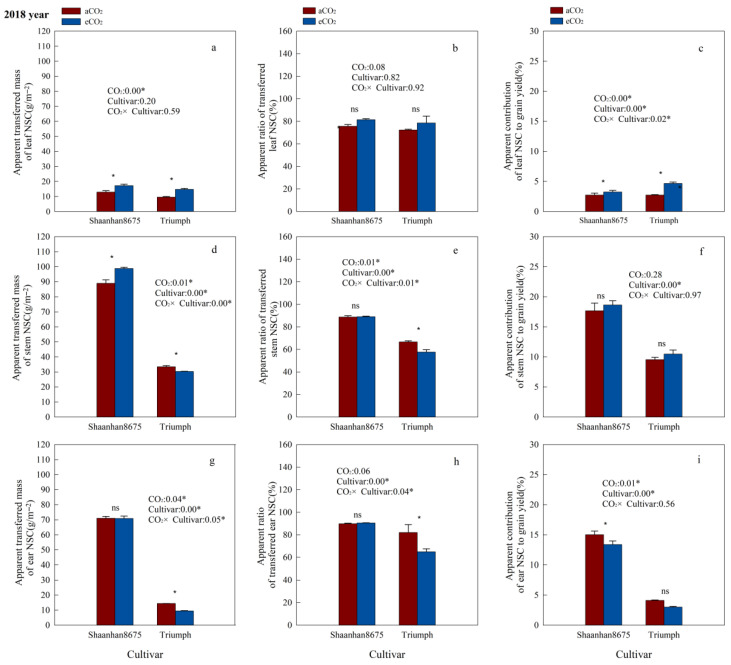
Effects of elevated CO_2_ on the ATM_NSC_ (apparent transferred mass of organ NSC), AR_NSC_ (apparent ratio of transferred NSC from organ), and AC_NSC_ (apparent contribution of transferred organ NSC to grain yield) of organs in two winter wheat cultivars grown under either ambient CO_2_ (aCO_2_, 415 µmol·mol^−1^) or elevated CO_2_ (eCO_2_, 550 µmol·mol^−1^) in 2018. Data represent the mean of three plants from each cultivar pot ± SD (standard error) bars. ANOVA results are also shown, with * indicating *p* < 0.05 and ns indicating no significance. (**a**) Apparent transferred mass of leaf NSC (g/m^−2^); (**b**) Apparent ratio of transferred leaf NSC (%); (**c**) Apparent contribution of leaf NSC to grain yield (%); (**d**) Apparent transferred mass of stem NSC (g/m^−2^); (**e**) Apparent ratio of transferred stem NSC (%); (**f**) Apparent contribution of stem NSC to grain yield (%); (**g**) Apparent transferred mass of ear NSC (g/m^−2^); (**h**) Apparent ratio of transferred ear NSC (%); (**i**) Apparent contribution of ear NSC to grain yield (%).

Elevated CO_2_ significantly increased the ATM_NSC_ and AC_NSC_ in leaves of Shaanhan8675 in both years (Figure 5a,c and Figure 6a,c), and significantly increased the ATM_NSC_, AR_NSC_, and AC_NSC_ in stems and ears of Shaanhan 8675 in 2017 (Figure 5a,c,d–i). However, except for the stem ATM_NSC_, which was significantly increased by eCO_2_ (Figure 6d), we did not observe any significant effect of eCO_2_ on NSC translocation parameters in the stems and ears of Shaanhan 8675 in 2018 (Figure 6e–h). Furthermore, eCO_2_ increased ATM_NSC_, AR_NSC_, and AC_NSC_ in the ears of Triumph by 145.3%, 114.1%, and 96.5% (*p* < 0.05) in 2017 (Figure 5g–i). In contrast, elevated CO_2_ in 2018 significantly decreased the ATM_NSC_ and AR_NSC_ in the ears and stems of Triumph (Figure 6d,e,g,h).

Additionally, we observed notable cultivar variations in NSC translocation parameters at the 550 µmol·mol^−1^ CO_2_ concentration. Shaanhan 8675 leaves had lower ATM_NSC_, AR_NSC_, and AC_NSC_ compared to Triumph in 2017 (Figure 5a–c). Nonetheless, the ATM_NSC_, AR_NSC_, and AC_NSC_ of Shaanhan 8675 stems and ears were higher than those in the stems and ears of Triumph in both years (Figure 5 and Figure 6).

### 3.7. Effects of Elevated CO_2_ on Grain NSC Accumulation at Ripening Stage

**Figure 7:**
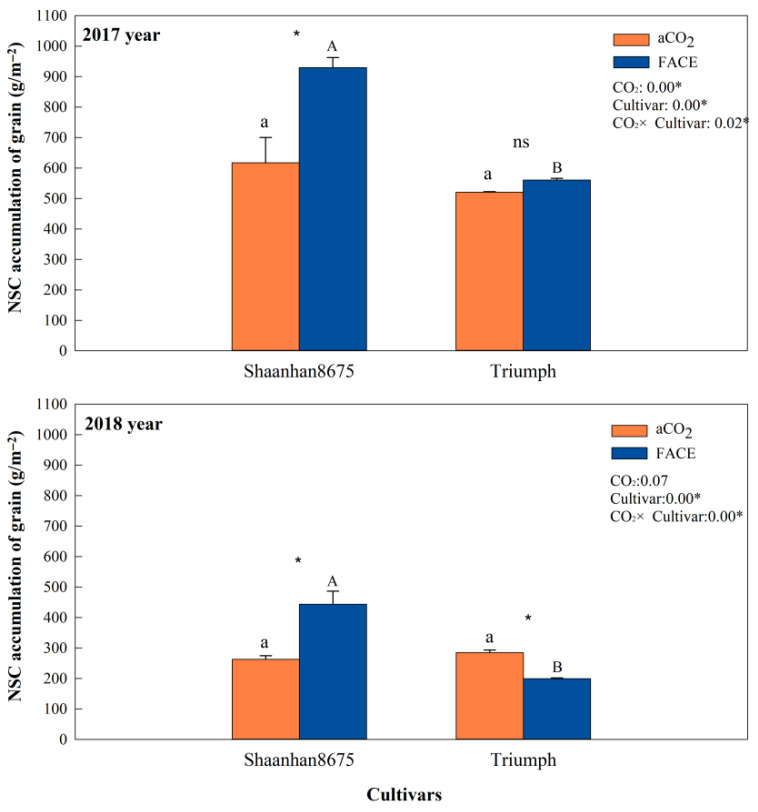
Effects of elevated CO_2_ on NSC (non-structural carbohydrate) accumulation of grain in two winter wheat cultivars grown under either ambient CO_2_ (aCO_2_, 415 µmol·mol^−1^) or elevated CO_2_ (eCO_2_, 550 µmol·mol^−1^) in 2017 and 2018. Data represent the mean of three plants from each cultivar pot ± SD (standard error) bars. Upper- and lower-case letters within each treatment indicate significant differences (*p* < 0.05) between cultivars under the eCO_2_ and aCO_2_ treatments. ANOVA results are also shown, with * indicating *p* < 0.05 and ns indicating no significance.

**Figure 8:**
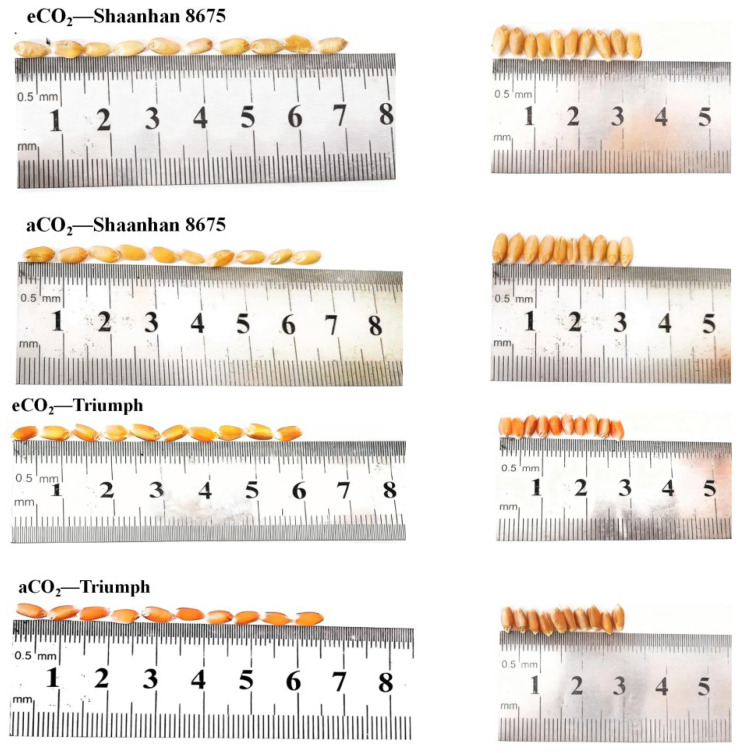
Effects of elevated CO_2_ on grain in two winter wheat cultivars grown under either ambient CO_2_ (aCO_2_, 415 µmol·mol^−1^) or elevated CO_2_ (eCO_2_, 550 µmol·mol^−1^). Grain photographs of Shaanhan 8675 and Triumph captured from horizontal and vertical angles.

Elevated CO_2_ increased grain NSC accumulation in Shaanhan 8675 by 50.6% and 69.0% across two years (Figure 7). Regarding the multiple-ear cultivar Triumph, eCO_2_ decreased grain NSC accumulation (30.0%, *p* < 0.05) in 2018. When CO_2_ concentration increased to 550 µmol·mol^−1^, the grain NSC concentration of Shaanhan 8675 was significantly higher than that of Triumph across two years. In Figure 8, the grain photographs of the two cultivars also demonstrate that elevated CO_2_ exerted significantly different effects on the two ear-type cultivars.

### 3.8. Correlation Analyses

**Figure 9:**
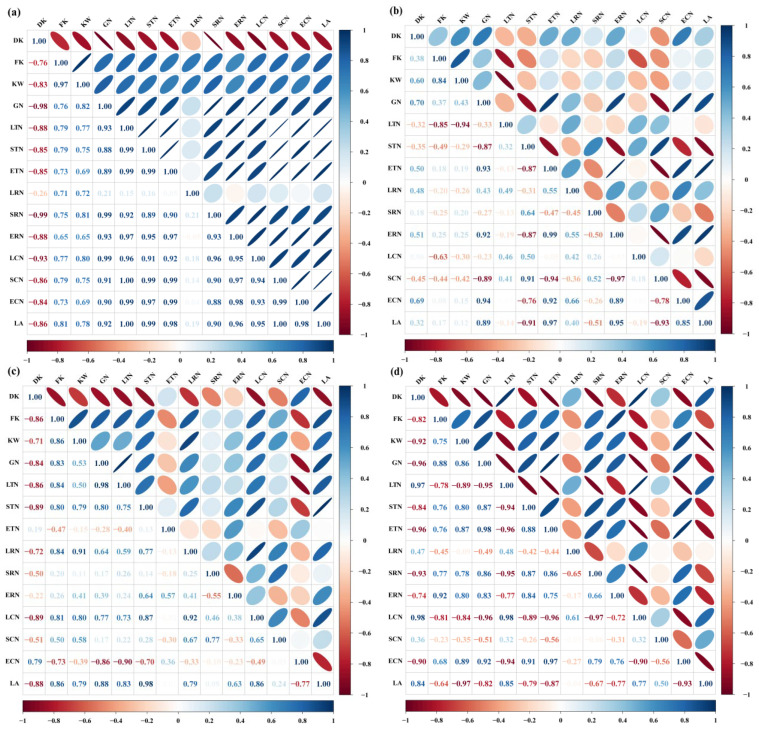
Correlation analysis between the grain NSC accumulation and NSC transfer-related parameters in 2017 (**a**,**b**) and 2018 (**c**,**d**). Positive values > 0.80 and negative values > −0.80 indicate a significant positive and negative correlation between the parameters, respectively. DK—degenerated kernels ear^−1^; FK—filled kernels ear^−1^; KW—kernel weight ear^−1^; GN—grain NSC accumulation; LTN—apparent transferred mass of leaf NSC; STN—apparent transferred mass of stem NSC; ETN—apparent transferred mass of ear NSC; LRN—apparent ratio of transferred NSC from leaf; SRN—apparent ratio of transferred NSC from stem; ERN—apparent ratio of transferred NSC from ear; LCN—apparent contribution of transferred leaf NSC to grain yield; SCN—apparent contribution of transferred stem NSC to grain yield; ECN—apparent contribution of transferred ear NSC to grain yield; LA—light-saturated net CO_2_ assimilation rate of flag leaves during the grain-filling period.

Elevated CO_2_ significantly enhanced the kernel weight of Shaanhan 8675 (Table 2). To identify the primary drivers behind this yield improvement, correlation analyses were conducted. The results revealed a significant positive relationship between kernel weight and grain NSC accumulation. Furthermore, grain NSC accumulation was positively correlated with the ATM_NSC_, AR_NSC_, and AC_NSC_ in various organs, as well as with the A_g,max_ of the flag leaf during the grain-filling period (Figure 9a,c). However, eCO_2_ did not exert a significant effect on the kernel weight of Triumph (Table 2). Correlation analysis indicated that grain NSC accumulation in Triumph was positively associated with the ATM_NSC_, AR_NSC_, and AC_NSC_ of the ear, as well as the A_g,max_ of the flag leaf during the grain-filling period. Notably, no significant correlation was found between kernel weight and grain NSC accumulation (Figure 9b). Grain NSC accumulation was positively correlated with the ATM_NSC_, AR_NSC_, and AC_NSC_ of the ear, as well as the ATM_NSC_ and AR_NSC_ of the stem (Figure 9d).

## 4. Discussion

Understanding how crop genotypes interact with rising atmospheric CO_2_ to modulate carbon metabolism and yield formation is central to breeding climate-resilient wheat [18]. Recent evidence highlighted that ear architecture critically influences grain yield potential, primarily by shaping sink strength and carbohydrate partitioning efficiency [19]. Large-ear cultivars, characterized by fewer but larger spikes with high assimilate demand per ear, were hypothesized to benefit more from CO_2_ fertilization due to their enhanced capacity for carbon accumulation and remobilization [20,21]. Using a two-year FACE experiment, we tested this hypothesis by comparing the physiological responses of a large-ear (Shaanhan 8675) and a multiple-ear (Triumph) wheat cultivar under ambient (aCO_2_, ~415 µmol·mol^−1^) and elevated CO_2_ (eCO_2_, ~550 µmol·mol^−1^).

### 4.1. The Effects of Elevated CO_2_ on Carbon Metabolism

The large-ear wheat cultivar Shaanhan 8675 exhibited consistent responsiveness to eCO_2_ across varying inter-annual meteorological conditions. Elevated CO_2_ significantly enhanced the *A_g_*_,_*_max_* and NSC accumulation in various organs of *Shaanhan 8675* over both experimental years. Concurrently, eCO_2_ significantly increased the SC in all organs, establishing a strong physiological basis for efficient NSC translocation [22]. Crucially, eCO_2_ upregulated SPS activity in Shaanhan 8675 leaves, promoting sucrose synthesis and facilitating phloem loading. Consequently, pre-anthesis NSC reserves, particularly in stems and ears, were efficiently remobilized during the grain-filling phase [23], as evidenced by significant increases in ATM_NSC_, AR_NSC_, and AC_NSC_. This robust source–sink coordination translated into 50.6% and 69.0% higher grain NSC accumulation, reduced spikelet abortion, and a 13.4% and 25.6% increase in kernel weight across years. These findings align with previous studies showing that eCO_2_ enhances carbon assimilation and carbohydrate accumulation in C_3_ plants [22], thereby promoting more effective carbon redistribution [24]. In contrast, Triumph exhibited highly variable responses contingent on inter-annual environmental conditions. In 2017, despite the moderate stimulation of *A_g_*_,_*_max_*, NSC accumulation in vegetative organs was limited. Instead, SC and the RS declined in leaves and stems, possibly because of feedback inhibition or greater respiratory carbon loss [25]. These effects may have been worsened by Triumph’s high tiller number and complex canopy. Sucrose serves as the main form of carbohydrate storage and transport in wheat organs [26]. Adequate sucrose levels are essential for maintaining osmotic balance during the translocation of carbohydrates from source to sink tissues [27]. Therefore, the ears of Triumph exhibited higher NSC translocation efficiency under eCO_2_ conditions. However, although ear RS increased, grain NSC accumulation and yield showed no significant improvement. In 2018, a year with higher precipitation during the grain-filling period, eCO_2_ failed to enhance flag leaf *A_g_*_,_*_max_* during the grain-filling period, and instead, reduced NSC translocation from stems and ears. This led to a 30.0% decline in grain NSC and a significant increase in degenerated kernels, ultimately decreasing kernel weight. The observed differences in *A_g_*_,_*_max_* between the two years may be explained by environmental variability. In 2018, the *A_g_*_,_*_max_* of Triumph flag leaves under aCO_2_ conditions was higher than that in 2017, resulting in a smaller relative enhancement under eCO_2,_ and thus, a nonsignificant difference when compared to 2018. This phenomenon could be influenced by higher precipitation during the grain-filling period in 2018 compared to 2017. Increased soil moisture can alter plant water use efficiency and indirectly affect leaf net photosynthetic rates [28]. For winter wheat grown under long-term eCO_2_ conditions, soil moisture status may modulate the magnitude of the CO_2_ fertilization effect [29]. Additionally, different cultivars exhibited varied responses to eCO_2_ and other environmental factors [30], which likely explains why the performance trend of Shaanhan 8675 differed from that of Triumph. The differing responses of Triumph across the two years highlight a key limitation of the multiple-ear strategy; although beneficial under favorable conditions, it is more sensitive to environmental variability under elevated CO_2_, likely due to poor coordination between source and sink tissues and less efficient carbon redistribution.

### 4.2. Intraspecific Variation in Response to Elevated CO_2_

Intraspecific variation in crop responses to eCO_2_ has been widely documented [6]. Our findings demonstrate that ear architecture fundamentally modulates wheat’s responsiveness to eCO_2_ through the integrated regulation of photosynthesis, NSC metabolism, and sink development. Under an eCO_2_ environment, Shaanhan 8675 consistently exhibited higher NSC and sucrose levels in stems and ears than Triumph, along with superior NSC translocation parameters (ATM_NSC_, AR_NSC_, and AC_NSC_). This finding aligns with previous studies showing that effective grain filling depends largely on the efficient transport of sucrose from source to sink tissues [31]. The greater increase in grain NSC concentration observed in Shaanhan 8675 compared to Triumph can be attributed to the superior NSC translocation efficiency in its stems and ears (Figure 6). Thus, efficient NSC transport within stems and ears appears to be a key determinant of improved grain-filling performance in wheat. This supports earlier findings that the remobilization of pre-anthesis NSC reserves stored in stems is a primary mechanism driving grain filling [32]. Moreover, prior research has confirmed that photosynthesis in the ears contributes significantly to the carbohydrate pool translocated to developing grains during the grain-filling period [33]. Under eCO_2_ conditions (550 µmol·mol^−1^), Shaanhan 8675 exhibited higher grain NSC concentrations than Triumph. Enhanced grain NSC accumulation facilitates the reduction in degenerated kernels and promotes the formation of more filled kernels per ear [34]. As a result, Shaanhan 8675 had fewer degenerated kernels and more filled kernels compared to Triumph (Table 2). The increased number of filled kernels contributed to Shaanhan 8675 achieving significantly higher kernel weight across both experimental years. Correlation analyses further clarified these divergences; in Shaanhan 8675, kernel weight was strongly and positively linked to grain NSC accumulation, which in turn correlated with the ATM_NSC_, AR_NSC_, and AC_NSC_ in the stems and ears and with the flag leaf *A_g_*_,_*_max_* during the grain-filling stage. In Triumph, however, no such relationship existed between the grain NSC and kernel weight in either year, implying a decoupling between carbon supply and grain-filling efficiency, likely due to insufficient sink strength or impaired translocation pathways. This physiological advantage facilitated more efficient carbon allocation to grains, reducing spikelet degeneration and increasing the number of filled kernels, both of which were key drivers of its yield gain. This conclusion corroborates previous reports showing that wheat cultivars with high kernel weight tend to store more NSC in stems before anthesis and exhibit higher NSC levels per spikelet [32].

### 4.3. Implications and Limitations

Collectively, our study provides compelling evidence that selecting large-ear wheat cultivars with strong sink capacity and efficient NSC remobilization traits can enhance yield stability and responsiveness under elevated CO_2_ scenarios. This supports a strategic shift toward optimizing ear architecture in breeding programs targeting climate resilience. It is important to acknowledge certain limitations of this study. A primary limitation lies in the methodology used to assess CO_2_ assimilation rates. In this work, net CO_2_ assimilation rates were measured using an LI-6400XT portable photosynthesis system (LI-COR Biosciences, Lincoln, NE, USA). However, as leaf respiration was not simultaneously measured, the data obtained reflect only net photosynthesis and not gross CO_2_ assimilation rates. This introduces a potential bias in our photosynthetic measurements and limits our ability to definitively determine which of the two wheat cultivars exhibited superior photosynthetic performance under elevated CO_2_ conditions. Future studies should, therefore, incorporate measurements of dark respiration to enable more accurate estimation of gross CO_2_ assimilation. Additionally, the number of wheat cultivars included in this study was limited. Only two cultivars, *Triticum aestivum* L. cv. Shaanhan 8675 (a large-ear type) and cv. Triumph (a multiple-ear type), was examined. The use of just one representative cultivar per ear type constrains the generalizability of the findings and may limit the representativeness of the results across broader genetic backgrounds. Despite these limitations, the current study provides valuable insights into how different ear types respond to elevated CO_2_, particularly in terms of NSC dynamics and grain filling. To build upon these findings, future research will focus on expanding the number of cultivars evaluated, including additional large-ear and multiple-ear wheat types, to better capture intraspecific variability and enhance the reliability and applicability of the conclusions.

## 5. Conclusions

In our case, the increase in grain NSC concentration and kernel weight of the large-ear wheat cultivar Shaanhan 8675 showed consistent stability across different inter-annual meteorological conditions. This consistency can be attributed to enhanced NSC accumulation and translocation in its vegetative organs under eCO_2_. Furthermore, eCO_2_ improved both the efficiency of NSC accumulation and its transport within Shaanhan 8675, which helped maintain a higher photosynthetic rate in the flag leaf during the grain-filling period. This sustained photosynthetic capacity contributed to more effective grain filling. Additionally, under eCO_2_ conditions, Shaanhan 8675 consistently exhibited higher grain NSC concentrations and kernel weights than Triumph across both experimental years. This was primarily due to greater NSC accumulation and more efficient redistribution from stems and ears in Shaanhan 8675 compared to Triumph. These results indicate that Shaanhan 8675 exhibited a positive physiological response to eCO_2_, particularly in terms of photosynthetic efficiency and carbon metabolism. In contrast, the multiple-ear cultivar Triumph showed variability in physiological responses between years, likely influenced by inter-annual climatic differences. These findings support our hypothesis that CO_2_ enrichment has a significant positive effect on NSC availability and remobilization from source organs to grains in the large-ear wheat cultivar Shaanhan 8675. This study provides valuable insights for future wheat breeding programs aimed at developing cultivars adapted to elevated CO_2_ environments. It highlights the potential to exploit the beneficial effects of climate change on wheat productivity through the selection and cultivation of genotypes with improved carbon utilization efficiency under future atmospheric conditions.

## Figures and Tables

**Table 1:** Ear traits and the ratio of harvest index of two cultivars.

Cultivar	Ear Number∙m^−2^	Ear Length (cm)	Stem Length (cm)	Grain Number per Ear	Grain Weight per Ear (g)	1000-Grain Weight (g)	HI
Shaanhan 8675	606.5	7.4	63.8	26.0	1.0	38.9	0.4
Triumph	733.7	6.8	90.7	20.5	0.7	33.1	0.3
ANOVA results	0.04 *	0.01 *	0.00 *	0.01 *	0.01 *	0.01 *	0.01 *

The value in this table is the average value of two harvests from 2016 to 2018. Significant effects of Cultivar are indicated as * (*p* < 0.05).

**Table 2:** Effects of elevated CO_2_ on the kernels of the ear and kernel weight at the ripening stage.

Cultivars	CO_2_ Treatments	Kernels Ear^−1^	Degenerated Kernels Ear^−1^	Filled Kernels Ear^−1^		Per Kernel Weight (mg)	Kernel Weight Ear^−1^ (mg)	Kernel Weight (g·m^−2^)
					2017			
Shaanhan 8675	aCO_2_	32.7 ± 1.0 ^a^	2.3 ± 0.2 ^a^	30.4 ± 0.9 ^a^		38.8 ± 1.4 ^a^	1180.8 ± 74.8 ^a^	865.1 ± 95.4 ^a^
	eCO_2_	34.9 ± 3.5 ^A^	1.7 ± 0.1 ^B^*	33.2 ± 1.9 ^A^*		40.1 ± 0.4 ^A^	1331.3 ± 90.8 ^A^*	978.9 ± 22.9 ^A^*
Triumph	aCO_2_	23.4 ± 0.3 ^b^	2.2 ± 0.3 ^a^	21.2 ± 0.2 ^b^		36.0 ± 0.8 ^a^	741.0 ± 9.9 ^b^	613.4 ± 27.1 ^b^
	eCO_2_	23.9 ± 0.4 ^B^	2.3 ± 0.1 ^A^	21.5 ± 0.4 ^B^		33.4 ± 1.9 ^B^	755.1 ± 35.8 ^B^	654.7 ± 16.3 ^B^
					2018			
Shaanhan 8675	aCO_2_	34.5 ± 0.6 ^a^	3.5 ± 0.4 ^a^	31.0 ± 0.5 ^a^		33.1 ± 0.2 ^a^	1023.8 ± 13.0 ^a^	450.0 ± 13.5 ^a^
	eCO_2_	36.7 ± 0.7 ^A^	2.7 ± 0.0 ^B^*	34.0 ± 0.6 ^A^*		34.4 ± 1.3 ^A^	1168.3 ± 52.4 ^A^*	620.3 ± 28.7 ^A^*
Triumph	aCO_2_	26.1 ± 1.1 ^b^	2.3 ± 0.1 ^b^	23.8 ± 1.1 ^b^		30.5 ± 1.2 ^a^	723.7 ± 4.4 ^b^	386.8 ± 8.7 ^a^
	eCO_2_	26.0 ± 0.1 ^B^	5.1 ± 0.6 ^A^*	20.9 ± 0.4 ^B^*		29.4 ± 1.3 ^B^	615.2 ± 27.7 ^B^*	346.3 ± 13.9 ^B^
ANOVA results	CO_2_	0.09 ^ns^	0.01	0.23 ^ns^		0.72 ^ns^	0.15 ^ns^	0.02
	Cultivar	0.00	0.00	0.00		0.00	0.00	0.00
	Year	0.01	0.00	0.22 ^ns^		0.00	0.00	0.00
	CO_2_ × Cultivar	0.14 ^ns^	0.00	0.01		0.08 ^ns^	0.01	0.02
	CO_2_ × Year	0.86 ^ns^	0.00	0.27 ^ns^		0.64 ^ns^	0.35 ^ns^	0.81
	Year × Cultivar	0.67 ^ns^	0.29 ^ns^	0.81 ^ns^		0.60 ^ns^	0.24 ^ns^	0.04
	CO_2_ × Year × Cultivar	0.84 ^ns^	0.00	0.21 ^ns^		0.65 ^ns^	0.40 ^ns^	0.23

Values are expressed as means ± standard error. aCO_2_—ambient CO_2_; eCO_2_—elevated CO_2_. Upper- and lower-case letters within each treatment indicate significant differences (*p <* 0.05) between cultivars under the eCO_2_ and aCO_2_ treatments. Significant effects of CO_2_ are indicated as * (*p* < 0.05). ANOVA results are also shown, with “ns” indicating no significance.

**Table 3:** Effects of elevated CO_2_ on NSC accumulation and partitioning index of plant tissues at the anthesis stage.

CO_2_ Treatments	Cultivars	NSC Accumulation (TM_NSC_ g·m^−2^)				NSC Partitioning Index (PI_NSC_)		
		Leaf	Stem	Ear		Leaf%	Stem%	Ear%
					2017			
aCO_2_	Shaanhan 8675	18.3 ± 0.4 ^b^	72.9 ± 5.3 ^a^	29.3 ± 0.7 ^a^		15.2 ^b^	60.4 ^a^	24.4 ^a^
	Triumph	26.2 ± 1.0 ^a^	83.2 ± 4.7 ^a^	24.6 ± 1.3 ^a^		19.6 ^a^	62.0 ^a^	18.4 ^b^
eCO_2_	Shaanhan 8675	26.2 ± 1.7 ^A^*	105.0 ± 7.4 ^A^*	60.5 ± 4.4 ^A^*		13.7 ^B^	54.7 ^B^*	31.6 ^A^*
	Triumph	23.9 ± 0.6 ^A^	82.4 ± 7.8 ^B^	28.2 ± 0.7 ^B^		17.9 ^A^	61.0 ^A^	21.1 ^B^
					2018			
aCO_2_	Shaanhan 8675	15.2 ± 0.7 ^a^	94.1 ± 5.1 ^a^	79.1 ± 1.0 ^a^		8.1 ^b^	49.9 ^b^	42.0 ^a^
	Triumph	13.2 ± 0.5 ^a^	50.1 ± 0.9 ^b^	17.8 ± 1.4 ^b^		16.3 ^a^	61.8 ^a^	21.9 ^b^
eCO_2_	Shaanhan 8675	19.2 ± 1.3 ^A^*	111.1 ± 1.3 ^A^*	78.2 ± 1.7 ^A^		9.2 ^B^	53.3 ^B^	37.5 ^A^*
	Triumph	18.9 ± 1.4 ^A^*	57.5 ± 2.8 ^B^	14.6 ± 0.7 ^B^		20.8 ^A^*	63.1 ^A^	16.1 ^B^*
ANOVA results	CO_2_	0.00	0.00	0.00		0.31 ^ns^	0.58 ^ns^	0.81 ^ns^
	Cultivar	0.27 ^ns^	0.00	0.00		0.00	0.00	0.00
	Year	0.00	0.05 ^ns^	0.00		0.00	0.07 ^ns^	0.00
	CO_2_ × Cultivar	0.01	0.01	0.00		0.12 ^ns^	0.49 ^ns^	0.23 ^ns^
	CO_2_ × Year	0.17 ^ns^	0.62 ^ns^	0.00		0.00	0.02	0.00
	Year × Cultivar	0.02	0.00	0.00		0.00	0.01	0.00
	CO_2_ × Year × Cultivar	0.00	0.12 ^ns^	0.00		0.19 ^ns^	0.14 ^ns^	0.37 ^ns^

Values are expressed as means ± standard error. aCO_2_—ambient CO_2_; eCO_2_—elevated CO_2_. Upper- and lower-case letters within each treatment indicate significant differences (*p* < 0.05) between cultivars under the eCO_2_ and aCO_2_ treatments; significant effects of CO_2_ are indicated as * (*p* < 0.05). ANOVA results are also shown, with “ns” indicating no significance.

**Table 4:** Effects of elevated CO_2_ on sucrose content and the ratio of sucrose to NSC at the anthesis stage.

CO_2_ Treatments	Cultivars	Sucrose Content (SC g·m^−2^)				Ratio of Sucrose to NSC (RS)		
		Leaf	Stem	Ear		Leaf%	Stem%	Ear%
					2017			
aCO_2_	Shaanhan 8675	4.0 ± 0.1 ^b^	26.8 ± 2.2 ^a^	5.7 ± 0.1 ^a^		22.1 ^b^	39.5 ^a^	19.6 ^a^
	Triumph	6.9 ± 0.3 ^a^	23.6 ± 1.1 ^a^	4.0 ± 0.2 ^b^		26.3 ^a^	28.5 ^b^	14.3 ^b^
eCO_2_	Shaanhan 8675	8.1 ± 0.6 ^A^*	50.9 ± 1.0 ^A^*	16.7 ± 0.8 ^A^*		31.0 ^A^*	45.4 ^A^*	27.9 ^A^*
	Triumph	3.7 ± 0.3 ^B^*	16.0 ± 0.9 ^B^*	4.5 ± 0.4 ^B^		16.5 ^B^*	19.5 ^B^*	18.2 ^B^*
					2018			
aCO_2_	Shaanhan 8675	1.9 ± 0.1 ^a^	22.8 ± 1.5 ^a^	12.5 ± 1.0 ^a^		12.6 ^b^	22.2 ^a^	15.8 ^a^
	Triumph	2.4 ± 0.1 ^a^	8.3 ± 0.3 ^b^	2.4 ± 0.1 ^b^		18.2 ^a^	16.5 ^b^	12.2 ^a^
eCO_2_	Shaanhan 8675	3.1 ± 0.2 ^A^*	40.4 ± 4.4 ^A^*	15.6 ± 0.1 ^A^*		16.1 ^A^*	36.6 ^A^*	19.9 ^A^*
	Triumph	1.8 ± 0.1 ^B^	5.6 ± 0.2 ^B^	2.7 ± 0.0 ^B^		8.7 ^B^*	10.8 ^B^*	18.6 ^A^*
ANOVA results	CO_2_	0.05	0.00	0.04		0.01	0.06 ^ns^	0.00
	Cultivar	0.00	0.00	0.00		0.00	0.00	0.00
	Year	0.00	0.00	0.21 ^ns^		0.00	0.00	0.00
	CO_2_ × Cultivar	0.00	0.00	0.08 ^ns^		0.00	0.00	0.01
	Year × CO_2_	0.61 ^ns^	0.54 ^ns^	0.50 ^ns^		0.03	0.00	0.06 ^ns^
	Year × Cultivar	0.25 ^ns^	0.02	0.01		0.00	0.17 ^ns^	0.01
	Year × CO_2_ × Cultivar	0.00	0.18 ^ns^	0.56 ^ns^		0.02	0.17 ^ns^	0.00

Values are expressed as means ± standard error. aCO_2_—ambient CO_2_; eCO_2_—elevated CO_2_. Upper- and lower-case letters within each treatment indicate significant differences (*p* < 0.05) between cultivars under the eCO_2_ and aCO_2_ treatments; significant effects of CO_2_ are indicated as * (*p* < 0.05). ANOVA results are also shown, with “ns” indicating no significance.

## Data Availability

The data presented in this study are available on request from the corresponding author. The data are not publicly available due to privacy and ethical restrictions.

## References

[1] Fahad S., Khan F.A., Pandupuspitasari N. (2019). Suppressing photorespiration for the improvement in photosynthesis and crop yields:A review on the role of S-allantoin as a nitrogen source. J. Environ. Manag..

[2] FAO (2016). The State of Food and Agriculture. Climate Change, Agriculture and Food Security.

[3] Li X., Khan A., Lv Z. (2019). Effect of multigenerational exposure to elevated atmospheric CO_2_ concentration on grain quality in wheat. Environ. Exp. Bot..

[4] Dier M., Sickora J., Erbs M. (2019). Positive effects of free air CO_2_ enrichment on N remobilization and post-anthesis N uptake in winter wheat. Field Crops Res..

[5] Urban O., Hlavacova M., Klem K. (2018). Combined effects of drought and high temperature on photosynthetic characteristics in four winter wheat genotypes. Field Crops Res..

[6] Thompson M., Gamage D., Ratnasekera D. (2019). Effect of elevated carbon dioxide on plant biomass and grain protein concentration differs across bread, durum and synthetic hexaploidy wheat genotypes. J. Cereal Sci..

[7] Asseng S., Kassie B.T., Labra M.H., Amador C., Calderini D.F. (2017). Simulating the impact of source-sink manipulations in wheat. Field Crops Res..

[8] Zhu C., Xu X., Wang D. (2015). An indica rice genotype showed a similar yield enhancement to that of hybrid rice under free air carbon dioxide enrichment. Sci. Rep..

[9] Marcos-barbero E.L., Pérez P., Martínez-carrasco R. (2021). Screening for higher grain yield and biomass among sixty bread wheat genotypes grown under elevated CO_2_ and high-temperature conditions. Plants.

[10] Lal M.K., Sharma N., Adavi S.B. (2022). From source to sink: Mechanistic insight of photoassimilates synthesis and partitioning under high temperature and elevated CO_2_. Plant Mol. Biol..

[11] Aranjuelo I., Cabrera-Bosquet L., Morcuende R., Perez P. (2011). Does ear C sink strength contribute to overcoming photosynthetic acclimation of wheat plants exposed to elevated CO_2_?. J. Exp. Bot..

[12] Córdoba J., Pérez P., Morcuende R. (2017). Acclimation to elevated CO_2_ is improved by low Rubisco and carbohydrate content, and enhanced Rubisco transcripts in the G132 barley mutant. Environ. Exp. Bot..

[13] Mustroph A., Boamfa E.I., Laarhoven L.J.J. (2006). Organ-specific analysis of the anaerobic primary metabolism in rice and wheat seedlings. I: Dark ethanol production is dominated by the shoots. Planta.

[14] Wang W., Li Q., Tian F. (2019). Wheat NILs contrasting in grain size show different expansion expression, carbohydrate and nitrogen metabolism that are correlated with grain yield. Field Crops Res..

[15] McCleary B.V., Charmier L.M.J., Mckie V.A. (2019). Measurement of starch: Critical evaluation of current methodology. Starch-Stärke.

[16] Pan J., Cui K., Wei D. (2011). Relationships of non-structural carbohydrates accumulation and translocation with yield formation in rice recombinant inbred lines under two nitrogen levels. Physiol. Plant..

[17] Yan B., Wu B., Gao Y. (2018). Effects of nitrogen and phosphorus on the regulation of nonstructural carbohydrate accumulation, translocation and the yield formation of oilseed flax. Field Crops Res..

[18] Cooper M., Voss-Fels K.P., Messina C.D., Tang T., Hammer G.L. (2021). Tackling G × E × M interactions to close on-farm yield-gaps: Creating novel pathways for crop improvement by predicting contributions ofgenetics and management to crop productivity. Theor. Appl. Genet..

[19] Zhang X., Jia H., Li T. (2022). TaCol-B5 modifies spike architecture and enhances grain yield in wheat. Science.

[20] Gaju O., Reynold M.P., Sparkes D.L. (2014). Relationships between physiological traits, grain number and yield potential in a wheat DH population of large spike phenotype. Field Crops Res..

[21] Lu D., Lu F., Pan J. (2015). The effects of cultivar and nitrogen management on wheat yield and nitrogen use efficiency in the North China Plain. Field Crops Res..

[22] Weichert H., Hoegy P., Mora-Ramirez I. (2017). Grain yield and quality responses of wheat expressing a barley sucrose transporter to combined climate change factors. J. Exp. Bot..

[23] Hu Y., Liu J., Lin Y. (2022). Sucrose nonfermenting-1-related protein kinase 1 regulates sheath-to-panicle transport of nonstructural carbohydrates during rice grain filling. Plant Physiol..

[24] Aranjuelo I., Erice G., Sanz-Saez A. (2015). Differential CO_2_ effect on primary carbon metabolism of flag leaves in durum wheat (*Triticum durum* Desf.). Plant Cell Environ..

[25] Adams M.W. (2018). Plant development and crop productivity. Handbook of Agricultural Productivity.

[26] Ma B., Zhang L., He Z. (2023). Understanding the regulation of cereal grain filling: The way forward. J. Integr. Plant Biol..

[27] Gong Z., Duan Y., Liu D. (2023). Physiological and transcriptome analysis of response of soybean (*Glycine max*) to cadmium stress under elevated CO_2_ concentration. J. Hazard. Mater..

[28] de Santana T.A., Oliveira P.S., Silva L.D. (2015). Water use efficiency and consumption in different Brazilian genotypes of *Jatropha curcas* L. subjected to soil water deficit. Biomass Bioenergy.

[29] Zheng Y.P., He C.L., Guo L.L., Hao L.H., Cheng D.J., Li F., Peng Z.P., Xu M. (2020). Soil water status triggers CO_2_ fertilization effect on the growth of winter wheat (*Triticum aestivum*). Agric. For. Meteorol..

[30] Zhang Y., Lam S.K., Li P. (2023). Early-maturing cultivar of winter wheat is more adaptable to elevated [CO_2_] and rising temperature in the eastern Loess Plateau. Agric. For. Meteorol..

[31] Yang J.C., Zhang J.H., Wang Z.Q. (2004). Activities of fructan-and sucrose-metabolizing enzymes in wheat stems subjected to water stress during grain filling. Planta.

[32] Liang W., Zhang Z., Wen X. (2017). Effect of non-structural carbohydrate accumulation in the stem pre-anthesis on grain filling of wheat inferior grain. Field Crops Res..

[33] Sánchez-Bragado R., Vicente R., Molero G., Serret M.D., Maydup M.L., Araus J.L. (2020). New avenues for increasing yield and stability in C3 cereals: Exploring ear photosynthesis. Curr. Opin. Plant Biol..

[34] Kashiwagi J., Yoshioka Y., Nakayama S., Inoue Y., An P., Nakashima T. (2021). Potential importance of the ear as a post-anthesis carbon source to improve drought tolerance in spring wheat (*Triticum aestivum* L.). J. Agron. Crop Sci..

